# Impact of planning horizon length on traits in an economic breeding goal and ranking of selection candidates in beef cattle

**DOI:** 10.1093/tas/txae090

**Published:** 2024-06-03

**Authors:** Hunter F Valasek, Ronald M Lewis, Bruce L Golden, Matthew L Spangler

**Affiliations:** Department of Animal Science, University of Nebraska, Lincoln, NE 68583, USA; Department of Animal Science, University of Nebraska, Lincoln, NE 68583, USA; Theta Solutions, LLC, Olympia, WA 98516, USA; Department of Animal Science, University of Nebraska, Lincoln, NE 68583, USA

**Keywords:** beef cattle, breeding objectives, economic selection index, planning horizon, relative emphasis

## Abstract

In beef production herds, unique situations such as breeding system, economic parameters, and current phenotypic performance can affect the emphasis of traits in the breeding goal and consequently the weighting of traits within a selection index. An often overlooked component of breeding goals is the planning horizon, or the time span to consider the economic impact of a selection decision, that varies between enterprises. A platform for constructing economic selection indexes (iGENDEC) was used to determine the impact of planning horizon length, breeding system, and sale endpoint on the relative emphasis of traits in the breeding goal and the re-ranking of selection candidates. As part of this investigation, the adjustment of phenotypic means for hot carcass weight and planning horizons were used to determine the impact of the relative emphasis on hot carcass weight as its mean approached a predetermined discount threshold. General-purpose indexes were created for animals sold at weaning and slaughter for three breeding systems with six different planning horizons (2, 5, 10, 20, 30, and 50 yr). As planning horizon increased, the relative emphasis on weaning weight or hot carcass weight, which affected revenue, decreased while the relative emphasis on stayability and mature weight increased. As the phenotypic mean for hot carcass weight approached and surpassed a predetermined discount threshold, the relative emphasis decreased before increasing again, once the mean weight surpassed the threshold. Rank correlations between indexes with different sale endpoints was 0.71 ± 0.1. Within a slaughter endpoint, re-ranking occurred between short and long planning horizons (*r* = 0.78 ± 0.09) while that of a weaning endpoint was less substantial (*r* = 0.85 ± 0.10). Jacard index scores between indexes with different planning horizons ranged from 39.7% to 87.9% and from 47.9% to 78.7% for weaning and carcass endpoints, respectively, for the top 5% of selection candidates. These results illustrate that the determination of a planning horizon can impact the rank of selection candidates and increases in net profit.

## Teaser

Planning horizon length and phenotypic means, particularly when pricing thresholds exist, to impact the relative emphasis of traits in breeding objectives. Incorrectly defining these parameters can lead to re-ranking of selection candidates.

## Introduction

Selection indexes were designed to aid in multiple-trait selection toward an aggregate goal (Hazel, 1943) and have become a vital tool when making selection decisions across a variety of species. Given the recognized economic criterion, selection indexes are utilized to maximize genetic improvement (Hazel et al., 1994). Selection indexes can generally be categorized by the breeding goal as either terminal or general purpose. General-purpose indexes offer the ability for self-replacing commercial herds to place emphasis on a combination of maternal and terminal traits. In U.S. beef production systems, cattle producers have multiple sale points at which they can market terminal offspring, with weaning and at the completion of the finishing phase being two common choices. Consequently, general-purpose indexes should incorporate the desired sale point of the terminal offspring (e.g., weaning or slaughter) conditioned on the identified breeding goal.

Explicit in the construction of economic selection index is obtaining, or estimating, genetic, phenotypic, and economic parameters, and current levels of performance (means) for the target (commercial) population. A large part in identifying a breeding objective is determining current phenotypic means within a herd as a basis for selection. The relative economic importance of traits is known to be impacted by differences in current levels of herd performance (Enns et al., 2006). Thus, there may be less economic incentive to increase a trait relative to other traits in the breeding objective if there is exceptional performance within the herd for that trait. This suggests that there is merit in the use of a non-linear profit function to determine the economic values of traits. Moreover, given the present genetic value of the animals within a herd, economic values should be calculated under the assumption that management decisions taken by the farmer maximize profit (Goddard, 1983).

Implicit in this exercise is defining the length of time from which to calculate the economic costs and returns from the genetic selection decision, herein called the planning horizon (PH). The choice of PH is complex and could be unique to individual enterprises as it contemplates the need for revenue at various points in the future by placing more or less relative emphasis on traits expressed earlier/later in life. Considering the initial financial situation, the PH is the given period to capture genetic and monetary returns, thus, creating a profitable sire selection program (McMahon et al., 1985). The consideration of a PH also impacts the number of expressions observed for traits. An increase in planning horizon resulted in an increased number of discounted genetic expressions across a variety of discounting rates before plateauing (Amer, 1999). The loss in expected number of expressions can be credited to the different points in life at which traits were expressed (i.e., reproductive longevity). While the idea of a PH is not new, there is little research on the impact this decision has on the relative emphasis of traits in a breeding objective and the ultimate ranking of selection candidates particularly in beef cattle populations. Given selection indexes are key to the selection decisions taken by beef producers, and offered broadly by U.S. beef breed associations, quantifying the impact of PH choice could inform revised indexes offered to U.S. beef producers.

The first objective of this current study was to investigate the impact of different lengths of PH, sale points, and breeding systems on the relative emphasis of traits in the breeding objective of beef cattle and the subsequent ranking of selection candidates. The second objective was to compare the relative emphasis when phenotypic means change for hot carcass weight (HCW) under different planning horizons.

## Materials and Methods

Approval from the Animal Care and Use Committee was not needed as the data used were simulated.

### Index Construction

This study was conducted using web-based software, iGENDEC, that allows for the construction of economic selection indexes in U.S. beef production systems (Spangler et al., 2022; https://github.com/blgolden/igendec) using the Beef Improvement Federation (BIF) implementation (http://igendec.beefimprovement.org/). General-purpose selection indexes, those intended for the production of both terminal animals and replacement females, were constructed for animals sold at weaning or slaughter under the assumption that replacement females were retained. Each point of sale included the following traits in the breeding objective and corresponding index: weaning weight-direct (WW-D), weaning weight-maternal (WW-M), mature cow weight (MW), stayability (STAY), heifer pregnancy (HP), calving ease-direct (CE-D), and calving ease-maternal (CE-M). The breeding objectives and indexes for animals sold at slaughter also included the traits: hot carcass weight (HCW), ribeye area (REA), fat depth (FAT), marbling score (MS), yearling weight (YW), and feed intake (FI). All traits were identified as economically relevant to the given breeding objectives. The trait of FI was considered as growing animal FI per day. Mature cow weight served as an indicator trait for mature cow feed intake but also a revenue generating trait through the sale of cull cows. The traits WW-D and YW were included for the slaughter endpoint to account for feed intake early in life and the value/cost of gain during this phase of production.

Given producer circumstances can vary, different situational indexes were constructed considering differences in breeding systems and planning horizon length. The indexes were constructed for three different breeding systems to investigate the impact of varying levels of direct (initial levels 0%, 50%, and 100%) and maternal heterosis (initial levels 0% and 100%). They included Angus bulls bred to Angus cows, F1 Simmental-Angus bulls bred to F1 Simmental-Angus females, and Simmental bulls paired with F1 Hereford-Angus females. The last component of index construction included six planning horizon lengths (2, 5, 10, 20, 30, and 50 yr) to reflect the varying number of years affected by improved genetic merit of bulls. The gradient of PH investigated enabled observations from a scenario akin to being terminal (i.e., PH = 2) to much longer PH whereby maternally expressed traits (e.g., STAY, MW) had an opportunity to be expressed through successive generations. Under the assumption that the economic parameters were the same for all scenarios described, there were a total of 36 indexes created (two sale points, three breeding systems, six PH). These parameters were the only aspects of index construction that were altered; thus, initial phenotypic means did not change based on breeding system or breed composition (Table 1).

**Table 1.** Phenotypic herd means for index construction.

Trait	Value	Units^1^
Calving loss due to dystocia	1.00	%
Conception rate	90.00	%
HCW	359.20	kg
REA	81.45	sq. cm
FAT	1.45	cm
MS	5.06	units
BW	38.56	kg
WW	247.35	kg
YW	385.55	kg
FI	11.34	kg/day
MW	589.67	kg

^1^Marbling score units where 5.0 = Sm^0^ and 6.0 = MT^0^.

HCW, hot carcass weight, REA, ribeye area, FAT, fat depth, MS, marbling score, BW, birth weight, WW, weaning weight, YW, yearling weight, FI, feed intake, MW, mature cow weight.

In addition to these indexes, the impact of increasing hot carcass weight towards a discount threshold was investigated. The value of HCW is non-linear in some U.S. markets such that animals above a given HCW threshold are discounted. Consequently, HCW represented an example to illustrate potential changes in breeding goal importance as phenotypic means change when revenue thresholds exist. The sensitivity of indexes to the mean of HCW was investigated using three PH (2, 20, and 50 yr) for the purebred Angus breeding system only. The three PH were chosen to reflect a short time horizon that would mimic a terminal system, a more intermediate PH, and a much longer PH such that the latter two enabled maternal traits to be more impactful. Given similarities in trends across breeding systems for the first objective, only one breeding system as considered. The discount threshold was predefined as 477 kg to represent a threshold weight that may exist in U.S. beef production systems. The mean HCW began at 295 kg and surpassed the discount threshold to a final mean of 522 kg with increments of approximately 45 kg. The initial phenotypic means for all other traits were held constant. For both steers and heifers, the price schedule was defined as 4.12 USD per kg for individuals less than 295 kg or greater than 477 kg, and 4.56 USD per kg for individuals within the 295 to 477 kg range.

The marginal economic value (MEV) for each trait in the breeding objective was calculated by creating a cow population through stochastic simulation via iGENDEC and perturbing the genetic values of bulls by one unit, one trait at a time, similar to approaches used by others (MacNeil et al., 1994). The genetic and residual correlations underlying the simulation are available at https://github.com/blgolden/igendec/blob/master/defaultMaster.hjson.

The economic parameters used were the default values available in iGENDEC. Since the traits in the breeding objectives and in the indexes were the same, the MEV was the weights in the indexes. The relative emphasis (RE) of each trait within the breeding objective was also calculated where RE was defined as the absolute value of the MEV multiplied by the genetic standard deviation then divided by the sum of this product for all traits (Ochsner et al., 2017a).

### Index Analysis

To evaluate the impact of differences between indexes, the indexes were applied to a group of selection candidates (*n* = 27,123) provided by the American Hereford Association (AHA). This list of selection candidates were chosen because AHA published genetic predictions for all goal traits simulated whereas other breeds did not. Moreover, the goal was to investigate changes in rank of selection candidates and selection decisions made considering differences in index weights created by differences in assumptions made during index construction (i.e., length of PH). Using different breeds as potential selection candidates to compare indexes would have no effect on the comparison given genetic predictions would change by a constant value (base adjustment and breed effect). Spearman’s rank correlations were then calculated in a pairwise fashion to capture changes in ranking across different PH, sale endpoints, and breeding systems. To better understand the impacts of re-ranking, the top 0.5%, 1%, and 5% of selection candidates were analyzed across the initial 36 indexes to inspect how selection decisions were impacted by the construction of individualized indexes. Like with the calculations of Spearman’s rank correlation, this evaluation was conducted in a pairwise fashion using a Jaccard index to evaluate the number of animals shared between two given indexes. A Jaccard index scores (*J*; Jaccard, 1901), given as a percentage from zero to a hundred were calculated as


$$\begin{array}{l} J=\frac{I_{c,j}}{T_{c,j}}\;\times 100 \end{array}$$

where *I*_*c,j*_ is the number of identically retained animals between the chosen index (c)and the *j*th index, and *T*_*c,j*_ is the total number of unique animals that were retained between the chosen index and *j*th index.

## Results and Discussion

### Impacts of Breeding Systems on Relative Emphasis

Despite breed differences and varying levels of direct and maternal heterosis, there were few differences in the RE for both weaning and slaughter endpoints. In the current study, traits in the economic goal can be assumed to be quite robust given that differences in RE between breeding system were minimal. Presumably the breed and heterosis differences were not great enough to impact the general trends in RE. The fact that initial means for fertility traits were high could have negated some of the potential benefits of heterosis in subsequent generations. In the subsequent results, the RE values were averaged across breeding systems due to similar trends as PH increased for both endpoints.

### Relative Emphasis of Traits for a Weaning Endpoint

**Figure 1.**
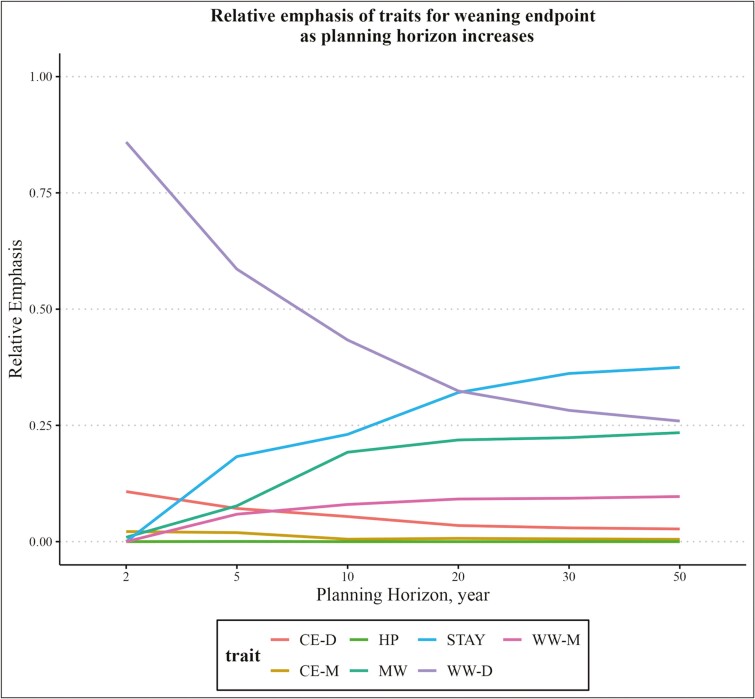
Comparison of relative emphasis, averaged over the three different breeding systems, for a weaning endpoint as planning horizon increases.

The RE values for WW-D decreased as STAY increased at a similar rate as PH shifted from 2- to 5 yr under a weaning endpoint (Figure 1). As PH continued to increase, these traits followed the same trend although with the rate of change decreasing before plateauing at the longer PH (30 or 50 yr). Mature cow weight also increased in relative emphasis as PH increased due to the ability to capture traits that were expressed later in an animal’s life. The relative emphasis of the other traits at the weaning endpoint (CE-D, CE-M, HP, and WW-M) were smaller in magnitude and less variable across all planning horizons. Regardless, given these traits represent identifiable sources of revenue or cost, their inclusion in a breeding objective and corresponding selection indexes is sensible. In the construction of a general-purpose index for U.S. Beefmaster cattle with a sale endpoint of weaning, the relative emphasis placed on WW-D (27.2%) and MW (49.2%) suggested that they were the most important traits (Ochsner et al., 2017b). However, Ochsner et al. (2017b) did not include STAY or specify a planning horizon. Moreover, the authors assumed different phenotypic means, particularly lighter weaning weights and greater incidence of calving difficulty, than assumed in the current study. In South African beef production, RE for WW-D ranged from 24.1% to 43.6% when bred to a specialized Angus sire (MacNeil and Matjuda, 2007). While those studies did not account for planning horizon, the RE values were consistent with those observed in the current study.

There is a relationship between the length of PH and the number of discounted expressions for traits expressed later in life, some of which could be profit drivers. While the breeds and relative emphasis of the traits varied, in the current study it was demonstrated that as PH horizon increased the RE increased for traits that are expressed later in life that drive profit within the weaning endpoint. This was because they had a greater opportunity to be expressed. Long-term planning horizons (~20 yr) were more desirable than short term due to the nature of livestock production and the impact on traits (Brash, 2002).

### Relative Emphasis of Traits for a Slaughter Endpoint

**Figure 2.**
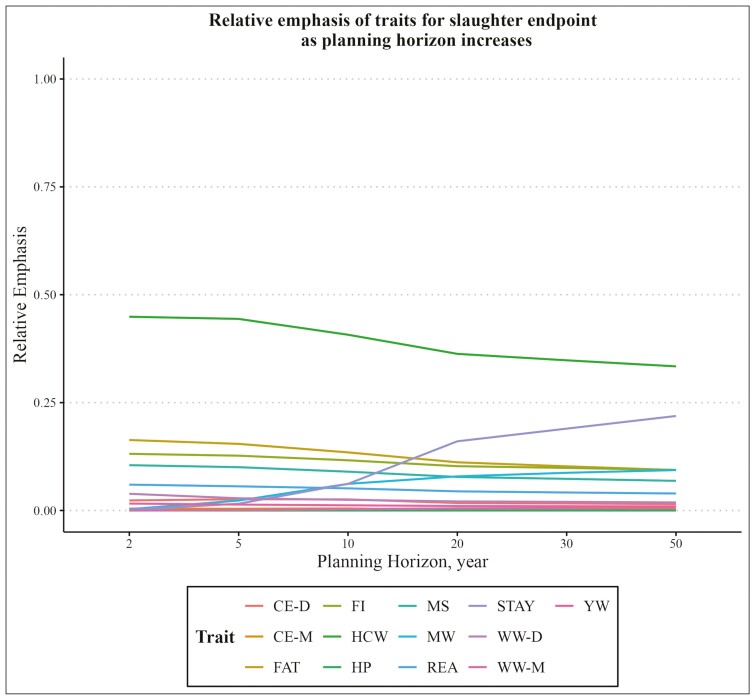
Changes in relative emphasis, averaged over the three different breeding systems, at a slaughter endpoint as planning horizon increases.

Similar trends to the weaning endpoint were observed between HCW and STAY for a slaughter endpoint (Figure 2). The relative emphasis for HCW steadily decreased while STAY increased as planning horizon increased. Economically, animals sold on a HCW basis would be more valuable (total revenue per animal sold) than animals sold at weaning, explaining why the RE of HCW could surpass that of STAY while the RE of WW-D would not. Therefore, HCW captured a significant portion of RE (>33%) across all planning horizons. An increase in relative emphasis for mature cow weight can be observed once again as PH increases, though it was less than when the endpoint was weaning. With shorter planning horizons, traits that are expressed later in life such as STAY and MW do not have the opportunity to be observed, thus traits expressed earlier in life are favored. The frequency and timepoint when traits are expressed within an animal’s lifetime differ among traits; thus, longer planning horizons allow for greater discounted genetic expressions of traits expressed later in life (Amer, 1999).

Similar relative emphasis values for carcass traits (HCW-59.5%, MS-11.1%, REA-5.5%, and FAT-4.6%) were used in the construction of a terminal index for Beefmaster cattle (Ochsner et al., 2017a). The variation in the relative emphasis values can be explained by the inclusion of maternal traits in the current study. Although the RE for some traits (e.g., carcass traits, some maternally expressed traits) in the current were relatively low, these traits are still economically relevant and merit inclusion in the breeding objective given they represent either a source of revenue or cost.

### Hot Carcass Weight Evaluation

**Figure 3.**
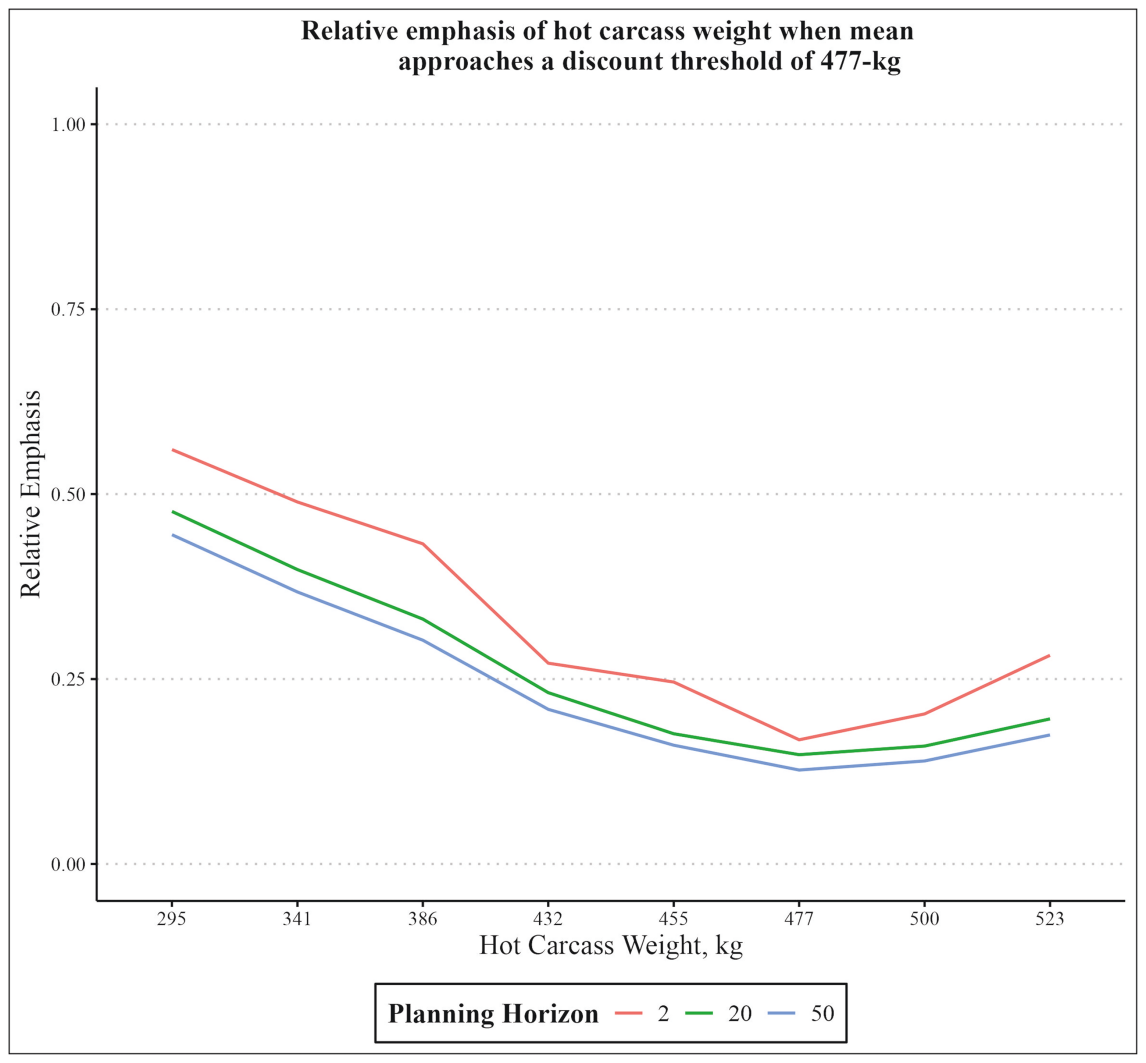
Relative emphasis of hot carcass weight for different hot carcass weight means and planning horizons when the discount threshold was 477 kg using a purebred Angus mating system.

For the HCW evaluation, changes in relative emphasis for HCW were observed as the herd mean for this trait changed across all three PH. The relative emphasis of HCW decreased as the mean herd HCW approached the discount threshold for all PH (Figure 3). The lowest relative emphasis for HCW, across all 3 PH, was observed when the mean HCW was equal to the discount threshold. Once the mean HCW surpassed the threshold the relative emphasis began to increase given the additional weight would counteract the revenue lost by the discount resulting in an increase of net profit. The RE of HCW was greatest for the 2-yr PH. The 20 and 50-yr PH has lower RE compared to the 2-yr. PH.

The differences between PH can be explained by the trends observed for RE at the slaughter endpoint (Figure 2). As PH increased, the emphasis on HCW decreased; thus, the results seen confirm that HCW captures less RE with longer PH (Figure 3). While this investigation did not test the lower limits of the pricing schedule, it can be inferred that the RE for HCW would have been greater than what was observed due to the need to increase mean HCW in the herd to not incur that discount.

The impacts of MW and STAY were investigated due to their increase in RE as PH horizon increased (Figure 2). The RE emphasis of MW never surpassed that of HCW for all three PH as the mean HCW increased and surpassed the discount threshold. For the 20-yr. PH, the RE of STAY surpassed, albeit slightly, that of HCW when the mean was 477 kg. When the PH was 50-yr. the RE of STAY surpassed that of HCW when the mean was > 432 kg (Figures 2 and 3). This is due to two primary reasons; there is greater economic incentive to place heifers in the feedlot because they are less likely to receive a heavy-weight discount and mature cows have heavier weight calves thus increasing total revenue.

### Re-ranking of Selection Candidates

Averaged over all PH and sale endpoints**,** there was little re-ranking of selection candidates when breeding system differed and the PH and sale endpoint remained the same (*r* = 0.96 ± 0.04). However, differences did exist between breeding systems when comparing shorter vs longer PH, particularly for the carcass endpoint. When comparing 2- or 5-yr PH to longer (20, 30, or 50 yr) PH rank correlations were consistently higher in the breeding system that utilized Simmental × Angus bulls mated to Simmental × Angus cows (*r* = 0.71 to 0.92). Rank correlations between 2- and 5-yr. PH and longer (20, 30, and 50 yr) PH were consistently lowest for the breeding system when Simmental bulls were mated to Hereford × Angus cows (*r* = 0.58 to 0.68). Crossbred females have an advantage in terms of sustained fertility, and placing selection pressure on additive genetic merit to improve this trait is not as important as it would be in purebred mating systems. Consequently, the MEV for STAY is not as large even in longer PH as might be the case in purebred mating systems. The lower rank correlations for the three-breed mating system simulated herein is an artifact of the introduction of a breed with lower breed effects for some carcass traits (e.g., MS and HCW). The MEV for these carcass traits changed to a greater degree between shorter and longer PH compared to the changes observed for the purebred and two-breed crossbreeding systems. Rank correlations between shorter and longer PH were more similar across breeding systems when the endpoint was weaning.

**Figure 4.**
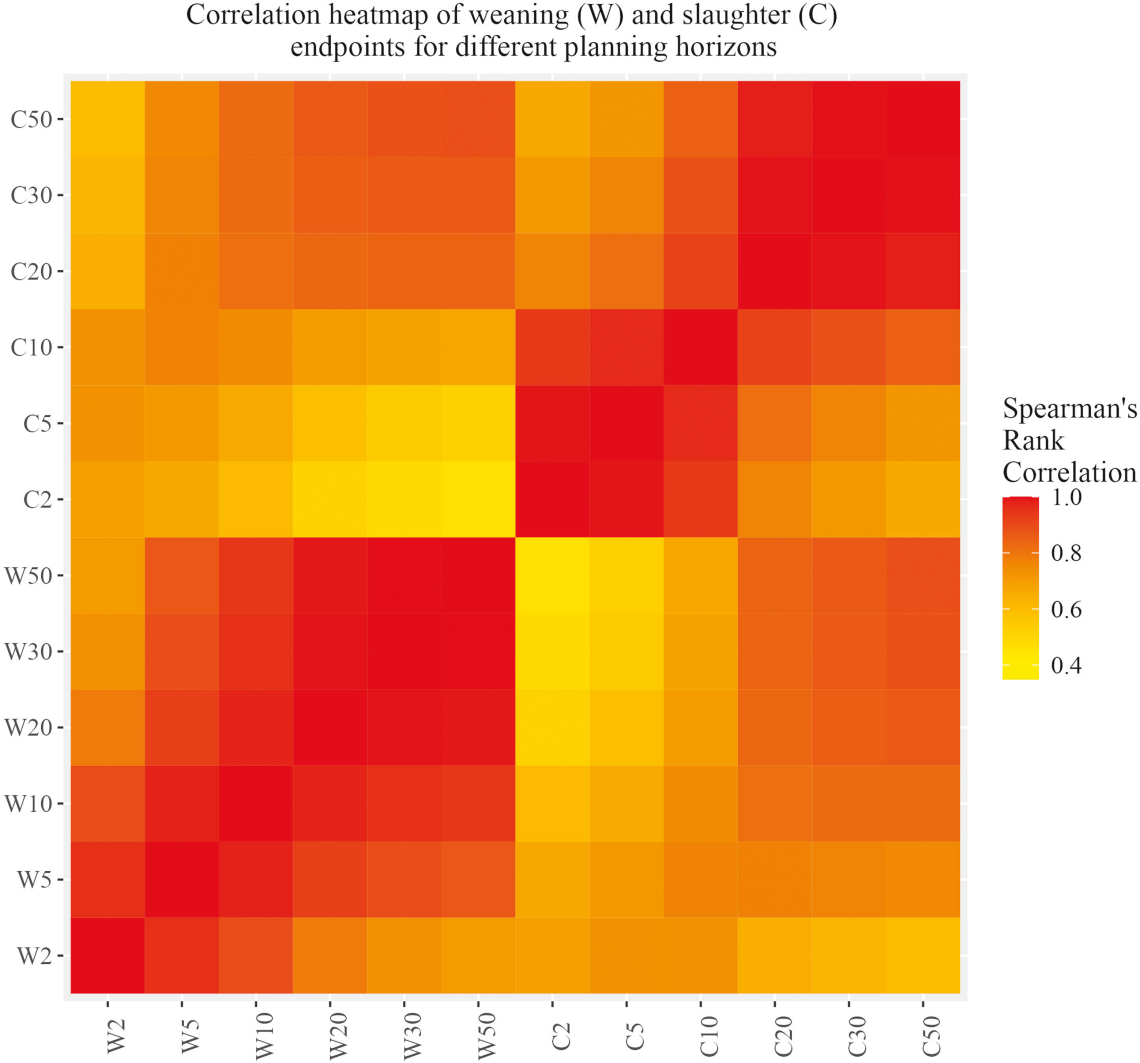
Heat map of Spearman’s rank correlation coefficients for weaning (W) and slaughter (C) sale endpoints for 2-, 5-, 10-, 20-, 30-, and 50-yr planning horizons averaged over the three breeding systems.

Averaged over breeding systems and within a slaughter endpoint, shorter PH (2, 5, and 10 yr) and longer PH (20, 30, and 50 yr) had re-ranking between them (*r* = 0.78 ± 0.09); however, the differences in rank correlations were less severe at a weaning sale endpoint (r = 0.85 ± 0.10) (Figure 4). When averaged over breeding system and PH, the average rank correlation coefficients between indexes of different endpoints were *r* = 0.71 ± 0.1. This result suggests that individuals rank differently depending on the point of sale of terminal offspring.

The re-ranking of top selection candidates, averaged across breeding system, was more substantial between PH of 2 and 5, 5 and 10, and 10 and 20 yr when the endpoint was weaning. The greatest re-ranking occurring between 10 and 20 yr PH when the endpoint was carcass. The extent of re-ranking depended on the sale endpoint of animals. On average for animals sold at weaning, there were 61.9 ± 15.5%, 65.6 ± 14.7%, and 73.1 ± 12.0% of selection candidates shared between two consecutive PH for the top 0.5%, 1%, and 5% of selection candidates, respectively (Table 2). There was a higher percentage of animals shared in the list of top selection candidates between consecutive PH when the endpoint was slaughter. For the top 0.5%, 1%, and 5% of selection candidates, two consecutive PH were found, on average, to share 68.9 ± 7.2%, 70.8 ± 7.4%, and 76.5 ± 6.7% of selection candidates (Table 2). Between the weaning and slaughter sale endpoints, 17.9 ± 3.7%, 21.8 ± 3.9%, and 33.5 ± 4.3% of selection candidates were shared, on average, between the same PH for the top 0.5%, 1%, and 5%, respectively (Table 2). As PH and the percentage of top selection candidates increased, the percentage of the same top selection candidates between two consecutive PH and sale endpoints increased. However, re-ranking was greater between endpoints and within a PH as compared to within an endpoint and between PH. Together these results suggest that choice of PH creates sensitivity in selection decisions and that selection decisions are more robust to additional extensions of PH once PH exceeds 20-yr. because all traits in the breeding goal have sufficient opportunity to be expressed. In a shorter PH (e.g., 2-5 yr) some traits are not expressed and as a consequence the MEV associated with them is negligible creating differences in the weighting of traits in the index.

**Table 2.** Percentage of top selection candidates in common between two consecutive planning horizons, as determined by a Jaccard index, within an endpoint and same planning horizon between endpoints

Endpoint^1^	Planning horizon(s)^2^	Top 0.5%^3^	Top 1%^3^	Top 5%^3^
Weaning	2 and 5	39.7 ± 20.9	43.8 ± 18.3	54.9 ± 15.7
	5 and 10	51.1 ± 25.0	56.4 ± 24.8	65.2 ± 20.3
	10 and 20	56.4 ± 11.8	58.6 ± 11.9	69.2 ± 8.7
	20 and 30	74.5 ± 4.6	76.1 ± 3.7	82.6 ± 3.1
	30 and 50	87.9 ± 4.3	92.9 ± 1.4	93.6 ± 1.2
Carcass	2 and 5	78.4 ± 5.3	78.0 ± 6.6	80.9 ± 5.4
	5 and 10	63.8 ± 12.9	62.3 ± 10.0	67.2 ± 11.7
	10 and 20	47.9 ± 10.6	52.6 ± 12.6	59.4 ± 10.4
	20 and 30	75.6 ± 4.8	77.2 ± 6.8	85.8 ± 5.2
	30 and 50	78.7 ± 2.1	83.9 ± 9.6	89.1 ± 1.0
Between endpoints	2	12.5 ± 2.6	12.8 ± 4.2	22.1 ± 6.9
	5	9.6 ± 3.7	11.1 ± 4.9	21.3 ± 7.5
	10	10.1 ± 4.1	15.1 ± 4.4	25.1 ± 5.1
	20	19.9 ± 2.7	24.7 ± 3.9	38.9 ± 4.0
	30	24.6 ± 4.5	30.6 ± 2.8	44.7 ± 0.7
	50	31.1 ± 4.9	36.5 ± 3.3	48.8 ± 1.7

Values are averaged over the three breeding systems.

^1^The point at which cattle are sold.

^2^Within endpoint, specifies the two planning horizons being compared.

^3^The percentage of animals with the highest index values out of the 27,123 selection candidates.

## Conclusions

The RE of traits for both weaning and slaughter sale endpoints indicated that traits expressed later in life (STAY and MW) were favored with longer-term planning horizons while traits reflecting revenue generated (WW-D and HCW) were favored with shorter-term planning horizons. These results suggest that a planning horizon length of approximately 20 yr is sufficient to balance RE across all traits in an index that assume replacements will be kept all other offspring marketed. However, there is no universally correct planning horizon choice when designing a breeding objective; there are additional considerations to be made. Such considerations include the desire or need for additional cash flow in the short term whereas a long-term PH may prioritize the long-term sustainability of the production system. Although several traits had lower RE values (carcass metrics, calving ease traits), these were clearly identified as economically relevant and as such should be included in the breeding goal. The choice of breeding system, including levels of heterosis and breed differences, should be considered in index construction given breed differences exist and gains made through exploiting heterosis could change the marginal economic value of changing traits through additive genetic selection. Additionally, HCW RE decreased as phenotypic means approached a designated discounting threshold but increased when surpassing the threshold; thus, updating breeding objectives and corresponding selection indexes as populations change is needed. Consequently, the current population phenotypic performance needs to be considered in index construction, and indexes updated as populations change. Lastly, selection candidate ranking was largely sensitive to sale endpoint of animals and the length of planning horizon within endpoints. The re-ranking of selection candidates confirms that utilizing and selecting upon one breeding objective is crucial in making accurate selection decisions for overall profitability.
